# Gastroschisis

**Published:** 2012-10-01

**Authors:** Vivek Gharpure

**Affiliations:** Department of Pediatric Surgery, Children's Surgical Hospital 13, Pushpanagari, Aurangabad, 431001, India

 (This section is meant for residents to check their understanding regarding a particular topic)

## Questions

1. What are the principles governing management of Gastroschisis?
2. What should be the mode of delivery if Gastroschisis is diagnosed antenatally?
3. What is Closed Gastroschisis?
4. What chromosomal anomalies are associated with Gastroschisis?
5. What preoperative preparation and monitoring is recommended for a newborn with gastroschisis?
6. What surgical adjuncts can be used to facilitate gastroschisis repair?
7. Discuss silo technique of gastroschisis closure?
8. What are gastrointestinal complications of gastroschisis?
9. What are sequelae of gastroschisis repair?
10. What is Plastic sutureless technique of gastroschisis closure?

## Answers

Surgical repair of abdominal wall defects involves replacing the abdominal organs back into the abdomen through the abdominal wall defect, repairing the defect if possible, or creating a sterile pouch to protect the intestines while they are gradually pushed back into the abdomen. Immediately after delivery, the exposed organs are covered with warm, moist, sterile dressings. A nasogastric tube is passed to keep the stomach empty and to prevent choking or aspiration. Under general anesthesia, an incision is made to enlarge the abdominal wall defect. The intestines are examined closely for signs of damage or additional birth defects. The organs are replaced into the abdominal cavity and the incision closed, if possible. If the abdominal cavity is too small or the protruding organs are too swollen to allow the skin to be closed, a pouch will be made from a sheet of plastic to cover and protect the organs. Complete closure may be done over a few weeks. Surgery may be necessary to repair the abdominal muscles at a later time. The infant's abdomen may be smaller than normal. Placing the abdominal organs into the abdomen increases the pressure within the abdominal cavity and can cause breathing difficulties or abdominal compartment syndrome. The infant may require ventilatory support for a few days or weeks [1-3].
Mode of delivery has not been found to change outcomes in babies with Gastroschisis or Exomphalos [1].
Spontaneous antenatal closure of abdominal wall defect in a baby with gastroschisis results in gangrene of outlying bowel and proximal bowel atresia. Sometimes the entire small bowel can be lost. These babies require total parenteral nutrition, bowel reconstructions and intensive management [2].
Trisomy 13 and Trisomy 18 are sometimes associated with Gastroschisis. As these chromosomal defects have a poor prognosis, treatment should be started only after adequate counseling of parents [1,2].
(a)Immediately cover herniated bowel with warm saline soaked pads and cover with a polythene bag to prevent hypothermia. (b) Put the baby on side to prevent traction on mesentery and ischemia. (c) The baby can be put in a polythene baby bag during transport. (d) Feeding tube is inserted and stomach is kept empty. (e) IV fluids are administered to correct shock, electrolytes and plasma depending upon patient's condition and availability, antibiotics are administered. Blood glucose is monitored and corrected. Metabolic acidosis may not require correction but should be monitored carefully. (f) Loops of intestine may require untwisting to avoid strangulation and gangrene. (g) If the abdominal defect is small as compared to mass of herniated bowel, it should be enlarged immediately to avoid vascular compromise of bowel. (h) Bladder is catheterized to monitor fluid therapy [3-5].
Abdominal wall stretching; evacuation of meconium from bowel by careful milking; not removing the covering peel [3].
This technique was described by Fisher et al in 1985. A spring loaded readymade transparent silastic silo is used to cover herniated bowel. Spring stays inside the peritoneal cavity and keeps the silo in place. Application of silo is done under sedation. Over next few days, bowel is gradually reduced and eventually, abdominal closure is achieved. This obviates multiple anesthesias, sutures between silo and abdominal wall, vascular compromise and compartment syndrome. Transparent silo allows bowel inspection. Patients who undergo silo closure have been described to have ileus for a longer duration compared to those who undergo primary closure [3,5].
Gastroesophageal reflux; necrotizing enterocolitis; intestinal failure; paralytic ileus; inguinal hernia [5, 6].
(a) Immediately abdominal compartment syndrome may occur with primary repair. (b) Bowel adhesions are seen in a third and many have to undergo surgery for adhesions. (c) Scar complications are common. (d) Absence of umbilicus may cause mental distress in childhood. (e) Growth and development is within normal limits. (f) Some patients with complicated gastroschisis who are on TPN for long duration are known to develop, liver complications related to TPN and may require liver transplant. (g) Inguinal hernia [4].
Umbilical cord is used to cover and close abdominal wall defect [7].

## Footnotes

**Source of Support:** Nil

**Conflict of Interest:** None
